# Supplementary material for: Asynchronous magnetic resonance elastography: Shear wave speed reconstruction using noise correlation of incoherent waves
Source: Magn Reson Med. 2022 Oct 27;89(3):990–1001. doi: 10.1002/mrm.29502 (PMC9792433; doi:10.1002/mrm.29502)
Supplement: Supplementary file 1 — DATA S1 MATLAB code used to generate the numerical simulations of Figure 1 [file MRM-89-990-s002.zip › k-Wave/helpfiles/bioheatExact.html]

bioheatExact :: Functions (k-Wave)


# bioheatExact

Compute exact solution to Pennes' bioheat equation in homogeneous media.

## Syntax

```
T = bioheatExact(T0, S, material, dx, t)
T = bioheatExact(T0, S, [D, P, Ta], dx, t)
```

## Description

`bioheatExact` calculates the exact solution to Pennes' bioheat equation in a homogeneous medium on a uniform Cartesian grid using a Fourier-based Green's function solution assuming a periodic boundary condition [1]. The function supports inputs in 1D, 2D, and 3D. The exact equation solved is given by

```
dT/dt = D * d^2T/dx^2 - P * (T - Ta) + S
```

where the coefficients are defined below. Pennes' bioheat equation is often given in the alternative form

```
P0 * C0 * dT/dt =  Kt * d^2T/dx^2 - Pb * Wb * Cb * (T - Ta) + Q

T:  temperature                     [degC]
C0: tissue specific heat capacity   [J/(kg.K)]
P0: tissue density                  [kg/m^3] 
Kt: tissue thermal conductivity     [W/(m.K)]
Pb: blood density                   [kg/m^3] 
Wb: blood perfusion rate            [1/s]
Ta: blood arterial temperature      [degC]
Cb: blood specific heat capacity    [J/(kg.K)]
Q:  volume rate of heat deposition  [W/m^3]
```

In this case, the function inputs are calculated by

```
D = Kt / (P0 * C0);
P = Pb * Wb * Cb / (P0 * C0);
S = Q / (P0 * C0);
```

If the perfusion coefficient `P` is set to zero, `bioheatExact` calculates the exact solution to the heat equation in a homogeneous medium.

[1] Gao, B., Langer, S., & Corry, P. M. (1995). Application of the time-dependent Green's function and Fourier transforms to the solution of the bioheat equation. International Journal of Hyperthermia, 11(2), 267-285.

## Inputs

|  |  |
| --- | --- |
| `T0` | matrix of the initial temperature distribution at each grid point [degC] |
| `S` | matrix of the heat source at each grid point [K/s] |
| `material` | material coefficients given as a three element vector in the form: `material = [D, P, Ta]`, where `D` \| diffusion coefficient [m^2/s]; `P` \| perfusion coefficient [1/s]; `Ta` \| arterial temperature [degC] |
| `dx` | grid point spacing [m] |
| `t` | time at which to calculate the temperature field [s] |

## Outputs

|  |  |
| --- | --- |
| `T` | temperature field at time `t` [degC] |

## See Also

`kWaveDiffusion`
